# Supplementary material for: Dimerization and dynamics of human angiotensin-I converting enzyme revealed by cryo-EM and MD simulations
Source: eLife. 2025 Sep 24;14:RP106044. doi: 10.7554/eLife.106044 (PMC12459953; doi:10.7554/eLife.106044)
Supplement: Supplementary file 1. [file elife-106044-supp1.pdf]

Table S1 List of buffer conditions used in DSF analysis of sACE.

Buffer pH	Tris	HEPES	Bis-Tris	MES	Imidazole	Citrate	Phosphate	MOPS
	7.5	6.8	5.8	5.5	6.2	5.5	5.8	6.5
	7.7	7	6	5.7	6.45	5.75	6.1	6.7
	7.9	7.2	6.2	5.9	6.7	6	6.4	6.9
	8.1	7.4	6.4	6.1	6.95	6.25	6.7	7.1
	8.3	7.6	6.6	6.3	7.2	6.5	7	7.3
	8.5	7.8	6.8	6.5	7.45	6.75	7.3	7.5
	8.75	8	7	6.7	7.7	7	7.6	7.7
	9	8.2	7.2	6.9	7.95	7.2	8	7.9
Bicine	7.6	7.8	8	8.2	8.4	8.6	8.8	9
Citrate-phosphate	4	4.5	5	5.5	6	6.5	7	7.5
Carbonate	6	6.3	6.6	6.9	7.2	7.5	7.8	8
Tricine	7.4	7.6	7.8	8	8.2	8.4	8.6	8.8

All buffers were tested in the presence and absence of 10 mM EDTA for DSF.
