# Supplementary material for: Dimerization and dynamics of human angiotensin-I converting enzyme revealed by cryo-EM and MD simulations
Source: eLife. 2025 Sep 24;14:RP106044. doi: 10.7554/eLife.106044 (PMC12459953; doi:10.7554/eLife.106044)
Supplement: Supplementary file 2. [file elife-106044-supp2.pdf]

Table S2 CryoEM data collection, refinement, and validation statistics				
**Data collection and processing**				
Grid preparation	Vitrobot	Chameleon		
Microscope	Titan Krios	Titan Krios		
Camera	Gatan K3	Gatan K3		
Automation software	SerialEM	Leginon		
Voltage (kV)	300	300		
Frames collected per micrograph	40	30		
Dose per frame (e-/Å^2)	1.5	1.76		
Total electron dose (e-/Å^2)	60	52.82		
Defocus range (μM)	0.7 to 1.5	0.6 to 2		
Total micrographs	3,653	18,281		
Initial particle images (no.)	1,171,502	10,708,232		
Pixel size (Å)	1.07	0.83 (0.41 raw)		
Structure	sACE-3.65	sACE-2.99	sACE-3.05	sACE-3.15
Final particle images (no.)	88,930	48,375	51,497	36,947
Symmetry imposed	C1	C1	C1	C1
Map resolution (Å)	3.65	2.99	3.05	3.15
FSC threshold	0.143	0.143	0.143	0.143
EMDB	EMD-45733	EMD-46581	EMD-46579	EMD-46574
**Refinement**				
Model resolution				
FSC 0.5	3.9 (4.1)[a]	3.2	3.2	3.4
FSC 0.143	3.6 (3.7)[a]	3.0	3.0	3.1
Sharpening B factor	95.7	80.8	84.3	79.4
Refinement package	PHENIX & COOT	PHENIX & COOT	PHENIX & COOT	PHENIX & COOT
Model composition				
Protein residues	2400	2400	2400	2400
Carbohydrates				
NAG	31	27	30	29
BMA	4	4	4	4
FUC	1	1	1	1
Total atoms	20044	19989	20031	20017
B factors				
Protein	81.4	93.4	91.6	109.0
Carbohydrate	130.7	152.1	146.8	161.6
RMS deviations				
Bond length	0.004	0.004	0.006	0.004
Bond angle	0.669	0.678	0.773	0.689

Ramachandran (%)				
Favored	93.49	94.28	93.95	94.20
Allowed	6.51	5.72	6.05	5.80
Outliers	0	0	0	0
**Validation**				
MolProbity score	2.50	2.34	2.43	2.33
Poor rotamers (%)	3.75	4.18	4.43	3.66
Clash score	13.33	9.08	10.21	9.70
Correlation coefficient	0.74	0.85	0.85	0.83
Cbeta outliers	0	0	0	0
CaBLAM outliers	3.93	3.55	3.93	3.51
PDB ID	9CLX	9D5S	9D5M	9D55
[a]Unmasked resolution is given in parentheses				
