# Supplementary material for: Dimerization and dynamics of human angiotensin-I converting enzyme revealed by cryo-EM and MD simulations
Source: eLife. 2025 Sep 24;14:RP106044. doi: 10.7554/eLife.106044 (PMC12459953; doi:10.7554/eLife.106044)
Supplement: Supplementary file 3. [file elife-106044-supp3.pdf]

Table S3 Summary for the conformational states of individual sACE domains in our all-atom MD simulation

Glycosylated ACE (>2 μsec)				
	A:ACE-N	A:ACE-C	B:ACE-N	B:ACE-C
run1	rapid closing (<0.1 μsec) and stay closed	rapid closing (<0.1 μsec) and stay closed	rapid closing (<0.1 μsec) and stay closed	rapid closing (<0.1 μsec) and stay closed
run2	rapid closing (<0.1 μsec) and stay closed	rapid closing (<0.1 μsec), then D3a slowly separated from D3b and D4 (~0.7 μsec) to open up and stay open	rapid closing (<0.1 μsec), then D1a slowly separated from D1b and D2 (~1.2 μsec) to open up and stay open	rapid closing (<0.1 μsec) and stay closed
run3	rapid closing (<0.1 μsec) and stay closed	D3a slowly separated from D3b and D4 to open up and stay open	rapid closing (<0.1 μsec) and stay closed	rapid closing (<0.1 μsec) and stay closed
run4	D1a separated from D1b and D2 to open up further and stay wider open	rapid closing (<0.1 μsec) and stay closed	rapid closing (<0.1 μsec), then open up (~0.7 μsec) by D1a separated from D1b and D2 and stay open	rapid closing (<0.1 μsec) and stay closed
Non-glycosylated ACE (>1.8 μsec)				
run1	rapid closing (<0.1 μsec) and stay closed	rapid closing (<0.1 μsec) and stay closed	rapid closing (<0.1 μsec) and stay closed	stay open
run2	persistent open even wider until ~0.77 μsec, then close and stay close	rapid closing (<0.1 μsec) and stay closed	rapid closing (<0.1 μsec) and stay closed	rapid closing (<0.1 μsec), then D3a slowly separated from D3b and D4 to open up and stay open
run3	rapid closing (<0.1 μsec), then D1a slowly separated from D1b and D2 (~0.4 μsec) to open up and stay open	rapid closing (<0.1 μsec) and stay closed	rapid closing (<0.1 μsec), then open up (~0.4 μsec) by D1a separated from D1b and D2 and stay open	rapid closing (<0.1 μsec) and stay closed
run4	rapid closing (<0.1 μsec), stay closed until ~1.5 μsec, then D1a separated from D1b and D2 to open up and stay open	stay open till ~0.9 usec then close and stay closed	rapid closing (<0.1 μsec) and stay closed	rapid closing (<0.1 μsec) and stay closed
ACE monomer				
run1	rapid closing (<0.1 μsec) and stay closed	rapid closing (<0.1 μsec) and stay closed		
run2	rapid closing (<0.1 μsec) and stay closed	rapid closing (<0.1 μsec) and stay closed		
run3	rapid closing (<0.1 μsec) and stay closed	rapid closing (<0.1 μsec) and stay closed		
