# Supplementary material for: Dimerization and dynamics of human angiotensin-I converting enzyme revealed by cryo-EM and MD simulations
Source: eLife. 2025 Sep 24;14:RP106044. doi: 10.7554/eLife.106044 (PMC12459953; doi:10.7554/eLife.106044)
Supplement: Supplementary file 4. [file elife-106044-supp4.pdf]

Table S4: summary of heterogeneity analysis

Cryosparc	A_N	A_C	B_N	B_C
0	nothing	open/close, open when N bends down towards it, closes when N bends away from it	nothing	open/close transition, opens when N bends towards it, closes when N bends away
1	open/close	open/close opposite of A_N	open/close opposite of A_N	open/close together with B_N
2	open/close	small open/close together with A_N	very small open/close	open/close
3	open/close	open/close together with A_N	small open/close	open/close opposite to A_C
4	open/close	open/close opposite of A_N	open/close together with A_N	small open/close together with B_N
CryoDRGN				
1	open/close	small open/close opposite A_N	small open/close	open/close
2	small density changes but no real motion	small density changes but not real motion	open/close	small density changes but no real motion
3	open/close	small density changes but no real motion	open/close together with A_N	small open/close opposite B_N
4	small density changes but no real motion	small density changes but no real motion	open/close	open/close together with B_N
5	small open/close	small density chnages but no real motion	small open/close together with A_N	small density changes but no real motion
